# Supplementary material for: Estimating the population size of female sex workers and transgender women in Sri Lanka
Source: PLoS One. 2020 Jan 15;15(1):e0227689. doi: 10.1371/journal.pone.0227689 (PMC6961924; doi:10.1371/journal.pone.0227689)
Supplement: S1 File — (DOCX) [file pone.0227689.s001.docx]

**S1. Multiplier-based population size estimates from service counts and unique objects**

**Table 1: Size estimates based on the multiplier data, FSW**

| Multiplier Methods | Colombo | Kandy | Galle |
| --- | --- | --- | --- |
| 1. Unique object |  |  |  |
| No. of unique objects distributed | 100 | 100 | 100 |
| Received unique object, IBBS (%, 95% CI) | 10.2  (5.5, 14.8) | 14.6 (10.8 18.4) | 18.5 (12.6, 24.3) |
| Population size estimate | 980 (676-1,818) | 685 (543-925) | 540 (412-794) |
| 2. Number of individual FSW who were clients of specific NGOs* | 1,400 | 150 | 550 |
| Reported being a client of a specific NGO in IBBS (%, 95% CI) | 31.4 (25.5, 37.4) | 11.0 (7.9, 14.1) | 40.5 (34.3, 46.8) |
| Population size estimate | 4,459 (3743-5,490) | 1,364 (1,064-1,899) | 1,358 (1,175-1,603) |
| 3. Number of individual FSW who received a condom from a specific NGO | 625 | NA | 455 |
| Reported receiving condoms from a specific NGO in IBBS (%, 95% CI) | 29.0 (23.5, 34.5) | 6.5 (4.4, 8.7) | 40.1 (33.9, 46.3) |
| Population size estimate | 2,155 (1,812 -2,660) | NA | 1,134 (983-1,342) |
| 4. Number of individual FSW who were escorted to an STI clinic from NGO records | 18 | NA | 18 |
| Reported being escorted to an STI clinic by the staff of a specific NGO in IBBS (%, 95% CI) | 21.9 (16.3, 34.5) | 4.2 (2.4, 6.0) | 35.5 (29.3, 41.9) |
| Population size estimate | 82 (52-110) | NA | 51 (43-62) |
| Median values | 1, 2, 3  2,155 (1,812-2,660) | Median not possible to  calculate | 1, 2, 3  1,134 (983-1,342) |

*Programme data were collected from the NGOs Abhimani in Colombo, Saviya Development in Galle and Laksetha Sahana Sewa in Kandy. In IBBS in each

**Table 2: Size estimates based on the multiplier data – Transgender women**

| Multiplier Method | Colombo | Jaffna |
| --- | --- | --- |
| 1. Unique object |  |  |
| N of unique objects distributed | 100 | 100 |
| Reported receiving unique object, IBBS (%, 95% CI) | 16.7 (12.2, 21.1) | 34.0 (26.7, 41.4) |
| Population size estimate | 599 (474-826) | 294 (242-375) |
| 2. Number of individual transwomen who were clients of an NGO | 300 | 90 |
| Reported being a client of an NGO in IBBS (%, 95% CI) | 60.3 (53.3, 67.2) | 76.9 (71.3, 82.5) |
| Population size estimate | 498 (446-563) | 117 (110-126) |
| 3. Number of individual transwomen who received a condom from an NGO | 275 | 45 |
| Reported receiving condoms from an NGO in IBBS (%, 95% CI) | 51.8 (44.8, 58.9) | 44.6 (37.6, 51.5) |
| Population size estimate | 531 (467-614) | 101 (87-120) |
| 4. Number of individual transwomen who were escorted to an STI clinic | NA | NA |
| Reported being escorted to an STI clinic by the staff of an NGO in IBBS (%, 95% CI) | 25.5 (18.8, 32.0) | 27.8 (21.1, 34.7) |
| Population size estimate | NA | NA |
| Indicator  Median values | 1, 2, 3  531 (467-614) | 1, 2, 3  117 (110-126) |

Programme data were collected from the NGOs Heart to Heart for Colombo, and Journey for Life in Jaffna.
