# Supplementary material for: Estimating the population size of female sex workers and transgender women in Sri Lanka
Source: PLoS One. 2020 Jan 15;15(1):e0227689. doi: 10.1371/journal.pone.0227689 (PMC6961924; doi:10.1371/journal.pone.0227689)
Supplement: Attachment — Submitted filename: Estimating the Population Size of Female Sex Workers and Transgender Women in Sri Lanka - Review Comments.docx [file pone.0227689.s004.docx]

Review: PONE-D-19-24615

**Estimating the Population Size of Female Sex Workers and Transgender Women in Sri Lanka**

**Introduction**

1. “In the latest IBBS carried out in 2015…” But further up you write “in 2017-2018 Integrated Biological and Behavioural Surveillance (IBBS) surveys”? Please clarify in the text when the latest surveys were carried out.

**Methods**

2. “In the absence of a gold standard method for estimating the size of a KP, it is recommended to implement multiple methods simultaneously to minimise potential bias resulting from a single method [6].” Suggest deleting – this is not pertinent to Methods and you already state similar in the Introduction.
3. “We also compared our estimates to the UNAIDS-recommended estimates for Asia and the Pacific, as a check on validity of our estimates” Being close to UNAIDS estimates does not make them valid. UNAIDS aggregate estimates are based on previous reported estimates and as such may be subject to similar biases as your or any other PSE activity. Rather, UNAIDS estimates may be useful to assess consistency of your estimates against regional UNAIDS estimates.
4. Please provide more detail about the unique object multiplier: How many days / weeks before the UO distribution? How many venues? How many objects?
5. Please provide more detail re the RDS: Sampling duration? Sample size? No. waves reached? Was convergence reached for receipt of the unique object? Were there bottlenecks re the unique object receipt? Similar for the NGO service / membership multiplier. Were eligibility criteria for the survey consistent with those for NGO service utilization / NGO membership / and UO receipt? Was the sampling area consistent? If you prefer describe this in Results.

**Results**

6. “shanti (9.6%),” Please explain what a Shanti is.
7. “Mobility was found at 20.6% of spots.” I don’t quite understand what that means, please rephrase?
8. “The relationship between the estimated number of FSW and the census population was strong (R2=0.449).” If you are using “strong” as a recognized category to interpret R2 values, please provide a reference. If this is your own opinion, just state the value and discuss the strength of the relationship in Discussion. (Same comment applies to the similar sentence for the TGW estimates: “The relationship between the estimated number of TGW and the census population was strong (R2=0.393).”)
9. “Adjustment for mobility gave a slightly lower average estimate of 183 (range: 149-217), while adjustment for “a hidden population” an estimate of 331 (269-380).” I’m not clear to which locality this estimate refers to – please clarify in the text?
10. If I read the text right you report the median of the various RDS based multiplier estimates? Please state all estimates, including the resulting median value.

**Discussion**

11. “The results provide an important point for macro- and micro-level planning of HIV services” Unclear?
12. “In seven cities in Cambodia in 2012, it was assessed using the capture-recapture method that there were 2,690 (95% CI 2,600 - 2,780) TGW, with the highest number in Phnom Penh (1380, 95% CI 1350-1410), and 115-425 in other cities [28].” Please add the relative PSE for these values.
13. Please discuss in greater detail the (very) large proportions of “hidden” KP, i.e. KP not attending venues. Do other studies report similarly large proportions?

**Additional comments:**

- I would not think that enumeration was used here which implies that you actually counted KP members at a subset of DS? Rather it seems you use key informants to give you’re their best guess. I suggest rephrasing methods description and terms used.
- While very true that no gold standard exists for PSE, the use of multiple methods does not mean that the resulting PSE necessarily improve in validity (that would only be the case if the various PSE are scattered both above and below the true PS value). If e.g., all methods used provide biased estimates what is the advantage of using multiple methods? I suggest discussing the merit of your approach in your Discussion section.
