# Supplementary material for: Estimating the population size of female sex workers and transgender women in Sri Lanka
Source: PLoS One. 2020 Jan 15;15(1):e0227689. doi: 10.1371/journal.pone.0227689 (PMC6961924; doi:10.1371/journal.pone.0227689)
Supplement: Attachment — Submitted filename: Response to reviewers.docx [file pone.0227689.s005.docx]

**Comments of the 1st reviewer**

| Comments of the first reviewer | Responses of the authors |
| --- | --- |
| 1. The “service multiplier” method is a redundant invention of epidemiology. It is mathematically identical to capture-recapture estimation, and should be treated as such. There is well-established literature and a rich set of estimation methods which should be used. | While we agree that these two methods are based on similar principles, they have been used separately to estimate the size of KPs. In epidemiological research and literature, the multiplier method has been used as a stand-alone method to estimate the size of KPs. Please see the paper that outlines PSE methods used in HIV surveillance in key populations (Abdul-Quader AS et al. Estimating the size of key populations: current status and future possibilities. Curr Opin HIV AIDS 2014, 9:107–114)  Please also see WHO guidelines on PSE published in 2010 where the capture-recapture and multiplier methods are described separately.  Our study utilised similar methods as the study published in:  [Quaye S](https://www.ncbi.nlm.nih.gov/pubmed/?term=Quaye%20S%5BAuthor%5D&cauthor=true&cauthor_uid=25704987), [Fisher Raymond H](https://www.ncbi.nlm.nih.gov/pubmed/?term=Fisher%20Raymond%20H%5BAuthor%5D&cauthor=true&cauthor_uid=25704987), [Atuahene K](https://www.ncbi.nlm.nih.gov/pubmed/?term=Atuahene%20K%5BAuthor%5D&cauthor=true&cauthor_uid=25704987), et al: Critique and lessons learned from using multiple methods to estimate population size of men who have sex with men in Ghana. [AIDS Behav.](https://www.ncbi.nlm.nih.gov/pubmed/25704987) 2015 Feb;19 Suppl 1:S16-23 |
| 2. Each individual estimate for each service count should be reported, along with proper confidence intervals or Bayesian credible sets. It is not clear how “confidence intervals” were estimated for the medians of those estimates. | Multiplier-based estimates of each service count and unique object are now available as a separate file in Appendix.  Confidence intervals of proportions were calculated in RDS-A and applied to the service counts. |
| 3. Evaluate whether your response data will support three- or four-source capture-recapture estimation from the service lists. The data will likely be adequate if each and every person contacted was asked about their use of each of the services. Multiple-source estimates will be more precise than multiple two-source estimates. | The method of estimation that we used does not include this approach.    Our population size estimation process relies on calculating and comparing estimates from multiple sources (service-based multiplier, unique object multiplier and mapping with enumeration). |
| 4. (Minor) Please provide a reference for the “Random Set Generator”. | It is a reference no. 17) Random Integer Set Generator. Available at <https://www.random.org/integer-sets/> Accessed on 26 July 2019. |
| 5. The midpoint (average) of the minimum and maximum estimates is not the mean population size, and use of the word “mean” is inappropriate. | We have used the expression “midpoint” to describe a value which is exactly in between the estimated minimum and maximum of population size. In this case, it is mathematically equivalent to the calculation of mean. |
| 6. For adjustment for those who did not use hotspots, how was p2 estimated? I suspect it was estimated from the RDS, but readers should not need to guess. | p2 was estimated from IBBS-based on RDS.  This is added to the manuscript in track changes. |
| 7. How do you justify the use of the adjective “strong” for R2 = 0.449? Less than half of the variance in KP size is explained by population size. This is a weak measure of the success of the estimation. | The R-squared was used to determine plausibility of relationship between general population census and number of FSW and TG. We agree that the quantifier “strong” is not appropriate in the results section.  This is now addressed in the text: only R-squared values are mentioned in the results and the plausibility is explained in the discussion. |

**Comments of the 2^nd^ reviewer**

| Comments of the second reviewer | Responses of the authors |
| --- | --- |
| “In the latest IBBS carried out in 2015…” But further up you write “in 2017-2018 Integrated Biological and Behavioural Surveillance (IBBS) surveys”? Please clarify in the text when the latest surveys were carried out. | By “the latest IBBS carried in 2015”, we meant the IBBS for which the results were published. In 2017-2018, we carried out PSE (results published in this manuscript) along with IBBS. The results of this IBBS have not yet been published. We corrected the text of the manuscript, so that it reads “In the IBBS carried out in 2015…” |
| **Methods** 2. “In the absence of a gold standard method for estimating the size of a KP, it is recommended to implement multiple methods simultaneously to minimise potential bias resulting from a single method [6].” Suggest deleting – this is not pertinent to Methods and you already state similar in the Introduction. | That is fine, we did so. |
| 3. “We also compared our estimates to the UNAIDS-recommended estimates for Asia and the Pacific, as a check on validity of our estimates” Being close to UNAIDS estimates does not make them valid. UNAIDS aggregate estimates are based on previous reported estimates and as such may be subject to similar biases as your or any other PSE activity. Rather, UNAIDS estimates may be useful to assess consistency of your estimates against regional UNAIDS estimates. | We agree on this argument and revised the text so that it reads now: “We also compared our estimates to the UNAIDS-recommended estimates for Asia and the Pacific, as a check on consistency of our estimates…” |
| 4. Please provide more detail about the unique object multiplier: How many days / weeks before the UO distribution? How many venues? How many objects? | This was explained in the manuscript:  Approximately 100 purses were distributed to KP members in the cities where IBBS were done. The number of unique objects distributed was determined by the number of contacts the distributing outreach organisations anticipated reaching within one week.  In this corrected version of the manuscript, we added that the distribution of unique objects was done two weeks before IBBS implementation.  The number of venues in the cities where unique objects were distributed ranged between 11-32. |
| 5. Please provide more detail re the RDS: Sampling duration? Sample size? No. waves reached? Was convergence reached for receipt of the unique object? Were there bottlenecks re the unique object receipt?  Similar for the NGO service / membership multiplier. Were eligibility criteria for the survey consistent with those for NGO service utilization / NGO membership / and UO receipt? Was the sampling area consistent? If you prefer describe this in Results | The following was described in the first version of the manuscript: The following number of participants were included in the surveys: 458 FSW and 254 TGW in Colombo, 360 FSW in Galle, 362 FSW in Kandy and 252 TGW in Jaffna. We now added other information requested.  The review of the convergence and bottleneck plots of the unique object receipt revealed no issues for FSW in Kandy and for TGW in Colombo and Galle. There were minor issues with the convergence for FSW in Colombo, which was reached in the last quarter of the sample. In the sample of FSW in Galle there was one seed-network with a bottleneck and convergence was reached in the last third of the sample.  Eligibility criteria for IBBS were consistent to the eligibility criteria for NGO service utilisation, as well as for unique object distribution (this is added to the Methods). Sampling areas were also consistent – NGOs whose data were sought operated at the levels of cities where IBBS was implemented. |
| **Results** 6. “shanti (9.6%),” Please explain what a Shanti is. | Shantis are small houses, usually made from pieces of wood, metal, or cardboard, in which poor people live, especially on the edge of a city. Some sex workers are living and operating from shantis. We added the explanation to the text. |
| 7. “Mobility was found at 20.6% of spots.” I don’t quite understand what that means, please rephrase? | This means that at 20.6% of hotspots respondents reported that they visited more than one spot on a peak day. This has been rephrased in the text.  Mobility is defined as follows in Methods: “KP individuals interviewed at hotspots during Level 2 activities were also asked about their mobility across spots (in terms of how many hotspots on average they visit on a peak day).”  It is also described in Methods, when describing the parameters of calculating S_2_ (estimated number of KP individuals adjusted for mobility). |
| 8. “The relationship between the estimated number of FSW and the census population was strong (R2=0.449).” If you are using “strong” as a recognized category to interpret R2 values, please provide a reference. If this is your own opinion, just state the value and discuss the strength of the relationship in Discussion. (Same comment applies to the similar sentence for the TGW estimates: “The relationship between the estimated number of TGW and the census population was strong (R2=0.393).”) | The R-squared was used to determine plausibility of relationship between general population census and number of FSW and TG. We agree that the quantifier “strong” is not appropriate in the results section.  This is now addressed in the text: only R-squared values are mentioned in the results and the plausibility is explained in the discussion. |
| 9. “Adjustment for mobility gave a slightly lower average estimate of 183 (range: 149-217), while adjustment for “a hidden population” an estimate of 331 (269-380).” I’m not clear to which locality this estimate refers to – please clarify in the text? | This refers to selected DSs (this is added to the text). |
| 10. If I read the text right you report the median of the various RDS based multiplier estimates? Please state all estimates,  including the resulting median value. | This is now available as a separate file (as an Appendix). |
| 11. “The results provide an important point for macro- and micro-level planning of HIV services” Unclear?. | Micro-level planning is enabled through identification of hotspots in selected DSs where outreach, community-based services can be organised and provided to KPs.  Macro-level planning is enabled through knowing the sizes of KPs at national and sub-national level, which helps to plan resources, set up programme coverage targets and raise funds for KP programmes.  This is described in the Discussion. |
| 12. “In seven cities in Cambodia in 2012, it was assessed using the capture-recapture method that there were 2,690 (95% CI 2,600 - 2,780) TGW, with the highest number in Phnom Penh (1380, 95% CI 1350-1410), and 115-425 in other cities [28].” Please add the relative PSE for these values | No data were given on the relative PSE in the report that is referenced. |
| 13. Please discuss in greater detail the (very) large proportions of “hidden” KP, i.e. KP not attending venues. Do other studies report similarly large proportions? | This is not an unexpected finding since respondent-driven sampling enables the access to those segments of a population that choose not to socialise at outside venues due to fears of disclosure of their behaviours, stigma, etc. We discuss this in the last paragraph of a Discussion.  We could not find similar data from other studies. |
| Additional comments: - I would not think that enumeration was used here which implies that you actually counted KP members at a subset of DS? Rather it seems you use key informants to give you’re their best guess. I suggest rephrasing methods description and terms used.  - While very true that no gold standard exists for PSE, the use of multiple methods does not mean that the resulting PSE necessarily improve in validity (that would only be the case if the various PSE are scattered both above and below the true PS value). If e.g., all methods used provide biased estimates what is the advantage of using multiple methods? I suggest discussing the merit of your approach in your Discussion section. | You are right in saying that we did not count KP members but interviewed key informants to give their best guesses. We took out “enumeration” from the text.  However, the term “enumeration” has been commonly used to describe the mapping approach (for example, please see the paper by Odek WO et al.  Estimating the Size of the Female Sex Worker Population in Kenya to Inform HIV Prevention Programming published in PLOS One in 2014.)  This has been discussed extensively in the Discussion, we believe. The purpose of triangulating data generated by multiple methods is to reduce the bias of a single method. |

**Reviewer 3**

| **Comments of the third reviewer** | **Responses of the authors** |
| --- | --- |
| While the multiplier method is well-received and widely used method for estimating population size of KPs, geographical mapping with enumeration has a lot of weaknesses and challenges as they do not always meet the statistical rigor. | We think that both methods have their advantages and disadvantages. As we wrote in a Discussion section, mapping with enumeration has been the commonest method used to estimate the size of KPs in Asia. It is suitable for settings where KP members socialize at outside venues. Also, mapping of hotspots has proven to be very useful for planning outreach activities at hotspots. However, corrections have to be made to adjust for a proportion of a population that is hidden. There are a number of peer-reviewed manuscripts published that describe the methods and results of mapping studies conducted globally. |
| Why having penetrative anal sex with was considered to as a necessary inclusion criteria. Would be good to have some clarification. | That is a common epidemiological definition of MSM, and has been used in the previous round of mapping and IBBS in Sri Lanka. |
| The authors selected higher proportion of DSs from high density category. Would not that generate some bias. I was not sure if that was taken into consideration when analyzing the data to estimate at the national level. Would be good to have some clarification. | We wanted to avoid a random sample having majority of rural DSs selected - that would bias our estimates. Most DSs have small population size; only a few are urban. For that reason, DSs were stratified into three categories. The sampling approach was taken into account during the analysis. We think that bias introduced with this sampling process is limited, as the number of selected DSs from each category is approximately proportionate to the total population size of the DSs in the category. |
| The authors used the Delphi method to arrive at national and provincial estimates. Delphi method can always be biased because of the biases and interest of those at the meeting. Not sure if these are addressed. | We described now in the Discussion the biases of the Delphi method.  Delphi technique is very often used when generating KP size estimates. The process of the Delphi technique was transparent (as described in the Methods) - it was ensured that each member of the panel was informed of the results of extrapolation of PSE data to the national level and of the results of each round of the Delphi process. The Delphi panel was composed of national experts. Part of the Delphi principle involves seeking consensus through an interaction among the experts composing a panel.  Finally, the estimated sizes of KPs are only the estimates, and we also provided the lower and upper plausibility bounds. |
